# Analytical Calculations of Scattering Amplitude of Surface Plasmon Polaritons Excited by a Spherical Nanoantenna

**DOI:** 10.3390/nano11112937

**Published:** 2021-11-02

**Authors:** Anton V. Dyshlyuk, Alexey Proskurin, Andrey A. Bogdanov, Oleg B. Vitrik

**Affiliations:** 1 Institute of Automation and Control Processes (IACP) FEB RAS, Far Eastern Federal University (FEFU) and Vladivostok State University of Economics and Service (VSUES), 690041 Vladivostok, Russia; anton_dys@mail.ru; 2 School of Physics and Engineering, ITMO University, 197101 St. Petersburg, Russia; alexey.proskurin@metalab.ifmo.ru (A.P.); a.bogdanov@metalab.ifmo.ru (A.A.B.)

**Keywords:** surface plasmon polaritons, SPP, nanoantenna, SPP excitation

## Abstract

Since surface plasmon polaritons (SPPs) are surface waves, they cannot be excited by an incident plane wave, because free-space photons do not possess a sufficient in-plane momentum. Phase matching between the incident light and SPP can be achieved using a high-refractive-index prism, grating, or nanoantennas. In this work, we found an expression for the amplitude of SPP excited by an arbitrary 3D current distribution placed near a metal interface. The developed method is based on the well-known technique used in waveguide theory that enables finding the amplitudes of waveguide modes excited by the external currents. It reduces the SPP excitation problem to the summation of the set of emitters. As a particular example, we considered a spherical dipole nanoantenna on a metal substrate illuminated by a normally incident plane wave. The analytical calculations were in good agreement with the full-wave numerical simulations.

## 1. Introduction

Surface plasmon polaritons (SPPs) are the phenomenon of a wave propagating along the interface between a metal or highly doped semiconductor and a dielectric, formed due to the interaction between the collective excitation of the electrons and the electromagnetic wave. First observed by R. Wood in 1901 [1], the anomalies in the reflectance spectra were explained in terms of surface waves by U. Fano in 1941 [2]. However, the origin of these surface waves was explained later by R. Ritchie in 1957 [3]. The localization of the SPP energy near the interface, which becomes extremely strong at the resonant frequency, makes SPPs promising for miniaturization of optical integrated circuits. For instance, the use of plasmonic waveguides enabled the squeeze optical guided modes at telecommunication frequencies down to the nanoscale [4,5]. Other applications include subwavelength imaging [6] and surface-plasmon-assisted commercially available quantum cascade lasers [7,8]. Tight spatial localization of light in plasmonic structures is accompanied by a large amplification of the incident field, which opens up exciting opportunities for biosensing [9,10,11], enhancement of light–matter interaction [12,13], nonlinear optics [14,15,16], and Raman spectroscopy [17,18]. The use of plasmonic films improved the efficiency of GaN-based LEDs [19,20,21]. Plasmon excitations have been observed and studied in both localized and extended structures.

One of the most promising applications of SPP is biosensing. Due to the strong localization at the metal interfaces, SPP is extremely sensitive to thin films of analytes or molecules attached to the interface. The refractive-index sensitivity reaches 10^6^–10^7^ nm/RIU [10]. SPR-based biosensors allow the label-free analysis of biomolecules’ interaction, which provides realtime measurement of the analyte concentration and kinetics, and thermodynamic binding parameters. SPP has been used in interaction studies and screening of various of analytes, including nucleic acids, proteins, carbohydrates, whole cells and receptors, which has led to applications in such fields as clinical diagnostics, the pharmaceutical industry, and military defense. For a more comprehensive discussion of the properties of SPPs, we refer the reader, for example, to the following monographs and reviews: [10,22,23,24,25,26,27]. 

SPP is a surface wave and, therefore, cannot be excited from free space by a plane wave because simultaneous conservation of energy and in-plane momentum is impossible in this case. Many techniques enable such phase matching. They include diffraction gratings [28], frustrated total internal reflections methods in the Otto or Kretschmann configurations [29,30] or use of the defects (holes, notches, grooves, etc.) that break in-plane translation symmetry [31,32,33]. Another opportunity for the excitation of SPPs is the use of dielectric or plasmonic nanoantennas [34,35,36,37]. The SPP excitation efficiency in this case can be significantly enhanced due to the nanoantennas’ resonances [38,39,40]. Moreover, the control over the multipole resonances of the nanoantennas allows dynamic control over the directivity of the excited SPPs [41]. This effect can be used for on-chip multiplexing of SPPs [42].

The problem of a plane wave scattering placed on a small particle on a substrate that supports surface wave propagation can be solved analytically using the dyadic Green’s functions [43,44,45,46]. Although this method is consistent with the experimental results and full-wave numerical simulations, it is quite tedious as it requires the calculation of Sommerfeld integrals. The corresponding calculations are performed in Appendix A.

Another approach based on the multipole decomposition was suggested [47] and subsequently used for calculation of the light scattering on spherical silicon nanoparticles [48]. This procedure allows taking into consideration arbitrary electric field distribution. Nevertheless, the suggested method is applicable only for far-field calculations, and it neglects the imaginary part of the substrate permittivity. Moreover, this approach utilizes the integration of the Green’s function using a decomposition over the basis of spherical functions. This technique also implies a number of technical difficulties.

In [49] we presented a simple alternative approach based on the reciprocity theorem to calculate the SPPs’ contribution to the total field scattered by an infinite rod of small radius on a metal substrate. This work is intended to extend the results obtained in [33] for a full 3D geometry. The modified method enabled calculation of the SPP amplitude when excited by an arbitrary 3D electric current distribution. It allowed us to bypass the calculation of complex integrals and reduce the problem to summation over a set of sources (in the case of a finite set of dipoles) or integration over all the external currents. Following the principles established in [33], our approach was based on the orthogonality of modes and the reciprocity theorem. To illustrate this method, we considered a single spherical nanoantenna over a metal surface illuminated by a normally incident plane wave. First, we derived and illustrated the analytical results and then we validated them by full-wave numerical simulations in COMSOL Multiphysics software.

## 2. Methodology

**Figure 1:**
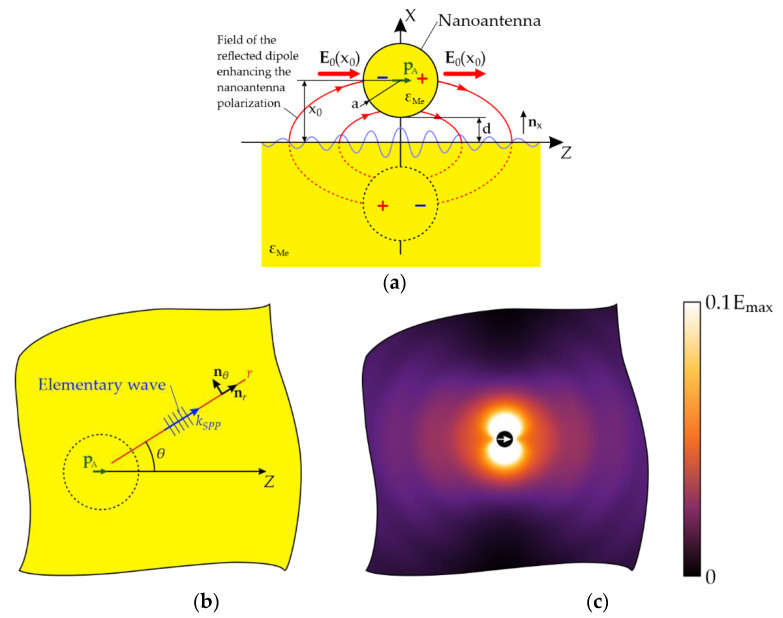
Excitation of the SPP wave on a metal film by a spherical nanoantenna. (**a**) Side view: the nanoantenna is affected by the field of the incident and reflected waves and the field due to the emission from the nanoantenna itself, the latter giving rise to the surface dressing effect (in the case of a perfectly conducting surface, it can be described as an impact of the mirror image shown dashed). (**b**) Top view: antenna and an elementary SPP wave excited by it on the surface of a metal film. (**c**) Amplitude of the continuous component of the electric field at x=0 (normalized to the field at r=0; λ = 550 nm, a = 50 nm, d = 10 nm).

Let us consider a nanoantenna: a small-radius sphere (*a* ≲ λ, where λ is the wavelength of light in the visible spectral range) made of a noble metal ($\varepsilon =\varepsilon_{Me}$) located in a vacuum (ε=1) at a distance *x*_0_ above the surface of a metal film and the gap between the sphere and the film is $d=x_{0}-a$. The film is assumed to be made of the same metal as the sphere, and it is sufficiently thick (thicker than the skin depth, which is about 15 nm for noble metals in the visible and near infrared spectral regions), and thus, that SPPs are excited only on its upper surface. The nanoantenna is illuminated by a linearly polarized plane wave incident vertically from above. Under this wave, the nanoantenna is polarized and acquires a dipole moment (DM) ***p*_A_**, which we assume to be parallel to the Z axis on the film surface (Figure 1).

We can represent the cylindrical surface plasmon-polariton wave excited by the nanoantenna as a set of plane waves propagating over the metal surface in all possible directions from the center of excitation, i.e., from the point in the ZOY plane just below the source. This approach is equivalent to the well-known method of decomposing an arbitrary wave into a spectrum of plane waves [50]. An alternative treatment using a decomposition over a set of cylindrical functions is provided in Appendix B. 

Each plane SPP wave representing an elementary component of this spectrum is the same TM mode as in the 2D case [51], but now it can propagate in any direction in the ZOY plane. The electric and magnetic fields of this elementary SPP wave are given by the following expressions:

$e=e_{0}e^{ik_{SPP}\left(z\cos\theta +y\sin\theta \right)}e^{-\gamma \left|x\right|}$, $h=h_{0}e^{ik_{SPP}\left(z\cos\theta +y\sin\theta \right)}e^{-\gamma \left|x\right|}$), where $e_{0}=\left(e_{0x}n_{x}+e_{0r}n_{r}\right)$, $h_{0}=h_{0}n_{\theta }$; $n_{x}$, $n_{r}$, $n_{\theta }$ are the basis vectors in cylindrical coordinates (Figure 1), $\varepsilon =\varepsilon \left(x\right)=\{\begin{array}{c} 1,\;x\geq 0\\ \varepsilon_{Me},\;x<0 \end{array}$ $e_{0x}=-h_{0}k_{SPP}{\left(\omega \varepsilon_{0}\varepsilon \right)}^{-1}$, $e_{0r}=ih_{0}\gamma_{D}{\left(\omega \varepsilon_{0}\right)}^{-1}$, $\gamma =\gamma \left(x\right)=\{\begin{array}{c} \gamma_{D},\;x\geq 0\\ \gamma_{Me},\;x<0 \end{array}$, $k_{SPP}=k_{0}{\left(\varepsilon_{Me}/\left(\varepsilon_{Me}+1\right)\right)}^{1/2}$ is the propagation constant of the elementary SPP wave, $\gamma_{D}=ik_{0}{\left(\varepsilon_{Me}+1\right)}^{-1/2}$, $\gamma_{Me}=ik_{0}\varepsilon_{Me}{\left(\varepsilon_{Me}+1\right)}^{-1/2}$, $k_{0}=\omega /c$, and $h_{0}$ is an arbitrary constant with A/m units (hereinafter, the factor $e^{-i\omega t}$ is implied but not written explicitly; for simplicity of formulae, we will also omit the x-dependence of ε and γ).

To calculate the spectral density of the plane wave decomposition of the entire surface plasmon-polariton wave excited by the nanoantenna, we used an expression derived from the non-conjugate reciprocity theorem [52]:

(1) $$\frac{da}{d\theta }=-\frac{1}{4N_{C}}\frac{1}{\lambda_{SPP}}\int_{V}e_{0}Je^{ik_{SPP}\left(z\cos\theta +y\sin\theta \right)}e^{-\gamma \left|x\right|}dV$$

where $N_{C}=\frac{1}{2}\left|\int_{-\infty }^{\infty }\left(e_{0}\times h_{0}\right)\cdot n_{r}dx\right|$ is the normalization of an elementary SPP wave; $\lambda_{SPP}=2\pi /k_{SPP}$ is the SPP wavelength, J is the current density in the nanoantenna, and *V* is the source volume. When deriving expression (1), we used the same approach as in [52], where the amplitudes of dielectric waveguides’ modes were calculated using the reciprocity theorem. However, since this approach implies no restrictions on the dielectric material conductivity, it can be applied to plasmonic waveguides as well.

If we assume that the radius of the sphere is infinitely small for simplicity, then the current density can be expressed as $J=i\omega p_{A}\delta \left(x-x_{0}\right)\delta \left(z\right)\delta \left(y\right)$. Equation (1) can then be integrated to yield $\frac{da}{d\theta }=A\cos\theta$, where $A=p_{A}\frac{\gamma_{D}\omega k_{SPP}}{2\pi a_{0}}\left|\frac{\varepsilon_{Me}^{3/2}}{\varepsilon_{Me}^{2}-1}\right|\exp(-\gamma_{D}x_{0})$. By integrating the contributions of all the elementary SPP waves diverging from the nanoantenna in the form $E_{\Sigma }=\int_{-\pi }^{\pi }\frac{da}{d\theta }e_{0}\cdot e^{i\beta \left(z\cos\theta +y\sin\theta \right)}e^{-\gamma \left|x\right|}d\theta$, we arrive at the following formula for the electric field of the total excited SPP wave: $E_{\Sigma }=E_{\Sigma r}n_{r}+E_{\Sigma \alpha }n_{\theta }+E_{\Sigma x}n_{x}$, where $E_{\Sigma r}=e_{0r}A_{SPP}\pi \left(J_{0}\left(k_{SPP}r\right)-J_{2}\left(k_{SPP}r\right)\right)e^{-\gamma \left|x\right|}\cos\theta$, $E_{\Sigma \theta }=-e_{0r}A_{SPP}\pi \left(J_{0}\left(k_{SPP}r\right)+J_{2}\left(k_{SPP}r\right)\right)e^{-\gamma \left|x\right|}\sin\theta$, $E_{\Sigma x}=2iA_{SPP}\pi J_{1}\left(k_{SPP}r\right)e^{-\gamma \left|x\right|}\cos\theta$. These expressions correspond to the components of a standing cylindrical wave. The reason for such a wave arising is that when the integration variable ranges from −π to π in the expression for $E_{\Sigma }$, each elementary wave propagated forward is combined with that travelling in the opposite direction. Separating from the total field the component corresponding to the wave diverging from the center and discarding the converging one, we obtain the final expressions for the components of the total SPP wave excited by the nanoantenna$\;E^{SPP}$:

$$E_{r}^{SPP}=iA_{SPP}e^{-\gamma \left(x\right)\left|x\right|}\left(H_{0}^{\left(1\right)}\left(k_{SPP}r\right)-H_{2}^{\left(1\right)}\left(k_{SPP}r\right)\right)\cos\theta$$

(2) $$E_{\theta }^{SPP}=-iA_{SPP}e^{-\gamma \left(x\right)\left|x\right|}\left(H_{0}^{\left(1\right)}\left(k_{SPP}r\right)+H_{2}^{\left(1\right)}\left(k_{SPP}r\right)\right)\sin\theta$$

$$E_{x}^{SPP}=-2A_{SPP}\varepsilon_{Me}^{\frac{1}{2}}\varepsilon {\left(x\right)}^{-1}e^{-\gamma \left(x\right)\left|x\right|}H_{1}^{\left(1\right)}\left(k_{SPP}r\right)\cos\theta$$

where $H_{m}^{\left(1\right)}$ are *m*-th order Hankel functions of the first kind, and the following factor independent of the coordinates:

(3) $$A_{SPP}=p_{A}k_{SPP}\gamma_{D}^{2}{\left(4\varepsilon_{0}\right)}^{-1}\left(\varepsilon_{Me}^{3/2}/\left(\varepsilon_{Me}^{2}-1\right)\right)\exp(-\gamma_{D}x_{0})$$

characterizes the excitation efficiency of the cylindrical surface plasmon-polariton wave by a dipole source. Using the asymptotics for the Hankel functions of a large argument, it is easy to show that the strength of the field component$\;E_{\theta }^{SPP}$ rapidly reduces to zero with increasing *r*, and at a distance of $\sim 2–3\lambda_{SPP}$ from the center of excitation, the total SPP wave has only the radial and vertical components. 

If it were not for the effect of the substrate (i.e., surface dressing effect), then in the framework of the electrostatic approximation, which is valid for a small-radius nanoantenna (*a* << λ), the magnitude of the dipole moment $p_{A}$ in Equation (3) would not differ from the dipole moment of a sphere in vacuum $p_{0}=a^{3}\varepsilon_{0}\left(\varepsilon_{Me}-1\right)E_{0}\left(x_{0}\right)/\left(\varepsilon_{Me}+1\right)$, polarized by the field of the incident wave and the wave reflected from the metal film surface: $E_{0}\left(x\right)=E_{0i}\left(e^{-ikx}+Re^{ikx}\right)\;$, where $R=\left(1-\sqrt{\varepsilon_{Me}}\right)/\left(1+\sqrt{\varepsilon_{Me}}\right)$ is the Fresnel reflection coefficient of the metal surface. To clarify the so-called surface dressing effect [43], let us assume for simplicity that the separated polarization charges in the sphere are point charges. Both these charges produce image charges in the metal film, which generate an inhomogeneous electric field (Figure 1). This induces both an additional dipole moment $p_{1}=qp_{0}$, where $q=\frac{\varepsilon_{Me}-1}{\varepsilon_{Me}+2}\frac{\varepsilon_{Me}-1}{\varepsilon_{Me}+1}\frac{a^{3}}{8{\left(a+d\right)}^{3}}$, and higher-order multipole moments of higher orders in the nanoantenna. The dipole moment $p_{1}\;$creates additional polarization charges in the nanoantenna, which, in turn, produce image charges in the film and induce an additional contribution to the dipole moment of the nanoantenna, and so on up to infinity. By summing up the resulting geometric progression, one can calculate a correction factor to $p_{0}$ taking into account the effect of the substrate: $\mu_{SDE}={\left(1-q\right)}^{-1}$. Another correction factor [53] $\mu_{a}={\left(1-\frac{3}{5}\frac{\varepsilon_{Me}-2}{\varepsilon_{Me}+2}{\left(ka\right)}^{2}-i\frac{2}{3}\frac{\varepsilon_{Me}-1}{\varepsilon_{Me}+2}{\left(ka\right)}^{3}\right)}^{-1}$ enables extending the validity of the electrostatic approximation for a sphere in vacuum making it applicable for relatively large spheres with radii corresponding to ka~1. Using both the correction factors, the dipole moment of the nanoantenna can be calculated as

(4) $$p_{A}=\mu_{SDE}\mu_{a}p_{0}$$

We note that the above expression is inaccurate for d≪a, since we are neglecting the image multipoles of higher orders for simplicity, which do affect the magnitude of the dipole moment of the nanoantenna at such small gaps between the sphere and the metal surface.

## 3. Results and Discussion

**Figure 2:**
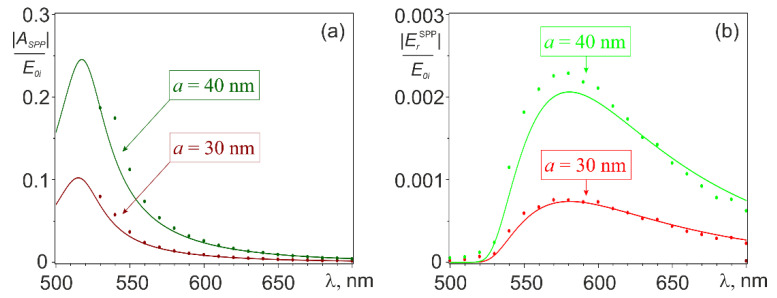
The efficiency (**a**) and amplitude at a distance of 8 μm from the excitation center (**b**) of the SPP wave vs. the incident wavelength, at d = 10 nm. Solid curves and dots are the results of the analytical and numerical calculations, respectively.

**Figure 3:**
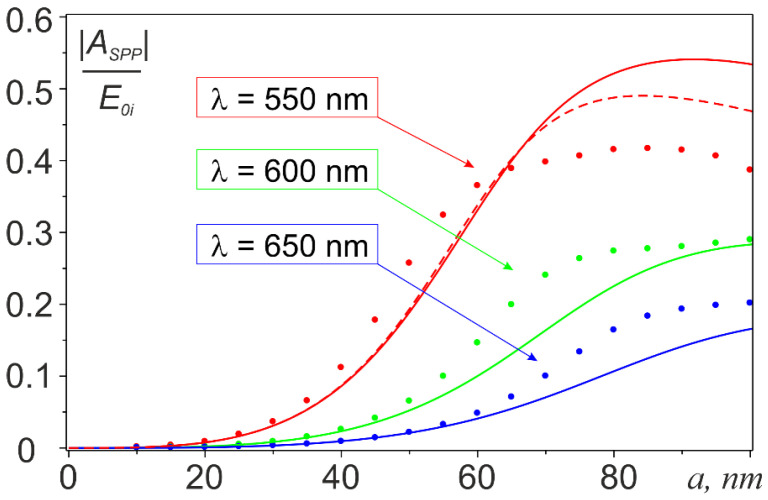
SPP excitation efficiency vs. nanoantenna radius at d = 10 nm. Solid curves are the results of analytical calculations; dots represent numerical data. The dashed line illustrates the correction of analytical results using the exact value of the dipole moment of the sphere in a vacuum.

**Figure 4:**
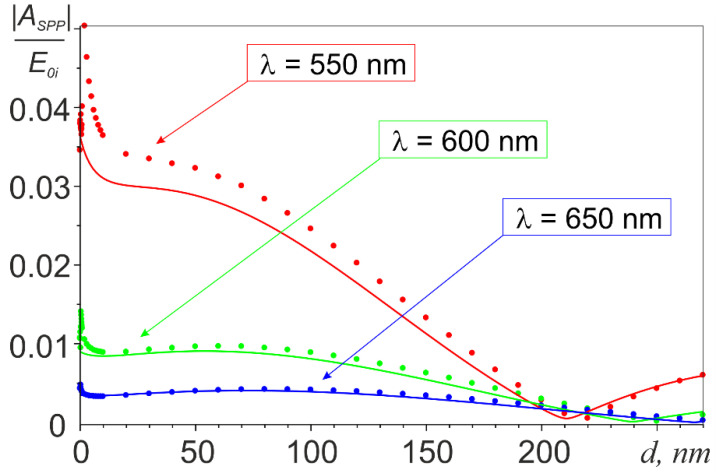
Dependences of the SPP excitation efficiency on the width of the gap between the nanoantenna and the film at a = 30 nm. Solid curves are the results of analytical calculations; dots represent numerical data.

The spatial distribution of the SPP wave excited on the film surface $\;E^{SPP}$ is illustrated in Figure 1c, which shows the pattern of its interference with the exciting field $E_{0}$ calculated with Equations (2)–(4). The results of calculating the SPP excitation efficiency *A_SPP_* are presented in Figure 2, Figure 3 and Figure 4 illustrating the effect of the incident wavelength (Figure 2), of the radius of the nanoantenna (Figure 3), and of the gap between the nanoantenna and the film (Figure 4). All calculations were performed assuming that both the nanoantenna and the film were made of gold, whose complex permittivity data were taken from the CRC reference book [54]. The lower limit of the spectral range for the calculations (*λ*_1_ = 490 nm) was chosen so that $Re\left(\varepsilon_{Me}\left(\lambda_{1}\right)\right)<-1$ was fulfilled in the entire spectral range of interest, it was the condition for SPP existence at the gold-vacuum boundary as a guided mode [55]. The upper limit of λ_2_ = 700 nm was set to keep the calculation range in the visible or, at most, in the near-infrared region. Note also that the results in Figure 2 and Figure 3 were obtained with a gap of d = 10 nm between the nanoantenna and the film. This was to minimize the effect of higher-order multipoles, which come into play at smaller gaps.

As can be seen from Figure 2a, the calculated dependences *A_SPP_(λ)* showed the maximum efficiency of SPP excitation, as expected, near the dipole resonance wavelength *λ_DR_* of the gold sphere (*λ_DR_* = 515 nm for the sphere with a radius of 30 nm and *λ_DR_* = 516 nm-for spheres with a slightly larger radius of 40 nm). It should be noted that the attenuation of the SPP wave at wavelengths close to *λ_DR_* was also very large [51]. The consequences of this are illustrated by Figure 2b, which represents the amplitude of the radial component of the SPP wave $E_{r}^{SPP}\left(\lambda \right)$, calculated using expression (2) at a distance of 8 μm from the excitation center (at *θ* = 0, *x* = 0). As one can see, for *λ ~ λ_DR_* the SPP amplitude became vanishingly small at this distance, which shifted the maximum of the amplitude to the red part of the spectrum, where the SPP attenuation was much smaller.

In Figure 3, we plotted the calculated dependences of *A_SPP_* on the nanoantenna radius solid curves. As one can see, the dependences first exhibited a growth according to the *a^3^* law, due to the corresponding increase in the nanoantenna dipole moment in the quasi-static approximation. The growth then slowed down and finally ceased at *a* ~ 0.15λ because of the influence of the correction factor *μ_a_* in expression 4. Prediction of the further variation of *A_SPP_* with *a* goes beyond our analytical approach. The main reason was not the limited applicability of the correction factor *μ_a_* (this could be amended by replacing the approximate value of the dipole moment of the sphere in a vacuum with its exact value known from the Mie theory), but it was no longer possible to neglect the effect of image multipoles in the film when the radius of the nanoantenna significantly exceeded the gap width *d*.

The effect of gap *d* on the efficiency of SPP excitation is illustrated by the solid curves in Figure 4. A sharp decrease in the SPP excitation efficiency was observed in the initial portion of the curves, which was explained by the rapid weakening of the effect of image charges in the film on the dipole moment of the sphere, until the latter, for gaps larger than a certain threshold value *d_SDE_*, approached the dipole moment of the sphere in a vacuum (the correction factor *μ_SDE_* in expression (4), which was responsible for this effect, rapidly decreased with increasing *d*/*a* ratio and became almost equal to unity at a gap of 0.27*a* for λ = 650 nm, 0.33*a* for λ = 600 nm, and 0.47*a* for λ = 550 nm). Figure 4 also shows that this effect was much more pronounced at a wavelength of 550 nm than at λ = 600 nm and λ = 650 nm. The reason was that a decreased coefficient *μ_SDE_* in expression 4 was partially compensated for by an increase in the amplitude of nanoantenna dipole moment. The latter moved from the minimum of the interference field E_0_ near the metal surface up to the maximum with increasing gap *d*. Calculations showed that |E_0_| increased faster with distance from the metal surface at longer wavelengths making the compensation effect more pronounced. At *d > d_SDE_*, the nanoantenna moved successively between the interference minima and maxima of the exciting field E_0_ with increasing *d*, which explained the further quasiperiodic character of the *A_SPP_(d)* dependence. The accompanying gradual decay of *A_SPP_*(*d*) was explained by the weakening effect of the nanoantenna on free electrons near the surface of the film, according to the law $e^{-\gamma_{D}x_{0}}$, as follows from expression (3).

To verify the analytical results, the problem of SPP excitation by a spherical nanoantenna was also solved numerically by the finite element method using COMSOL Multiphysics software. To simplify the calculations, the 3D geometry of the problem was reduced to a 2D axisymmetric geometry. The model contained a single half-plane parallel to the electric field of the incident wave and passing through the center of the nanoantenna. This half-plane was discretized by a nonuniform mesh with sufficiently small (to achieve convergence of numerical results) element size in the region of the gap between the metal surface and the nanoantenna. Absorbing boundary conditions (perfectly matched layers) were applied to the outer boundaries of the computational domain.

The calculated electromagnetic field near the nanoantenna apart from the obvious contribution of the exciting wave field (which was easily subtracted) consisted of the near and far field of the nanoantenna and the excited SPP wave. The amplitude of the first two components representing an unwanted background to the SPP wave of interest rapidly decreased with distance from the source at all wavelengths in the simulated spectral range. The SPP wave also decayed quickly with distance from the nanoantenna at shorter wavelengths, where it had high propagation losses. At longer wavelengths, these losses became lower at longer wavelengths, so the SPP wave contribution dominated at a relatively large distance from the excitation center. The numerical simulations showed that the amplitude of the longitudinal component of the electric field amplitude on the interface corresponded to the form r−1/2exp(−Im(kSPP)r) at λ ≳ 530 nm and *r* ≳ 10λ. This dependence was consistent with the asymptotic behavior of the Hankel functions in $\left|E_{r}^{SPP}\left(r\right)\right|$. That means that under the above conditions, the surface plasmon wave did in fact dominate in the total field at the metal surface. This conclusion enabled one to extract from the numerical simulation all the components of $\;E^{SPP}$. The corresponding results for $\left|E_{r}^{SPP}\left(r\right)\right|$ obtained at *r* = 8 μm, *θ* = 0, *x* = 0, and *d* = 10 nm are shown by dots in Figure 2b. They were consistent with the results of the analytical prediction. However, the agreement was better at *a* = 30 nm than at *a* = 40 nm. This could be explained by the neglected effect of image multipoles in the film. Considering the known law of SPP propagation, the obtained numerical results could be used to calculate the excitation efficiency *A_SPP_*, which is shown by dots in Figure 2a. As seen from the figure, the numerical and analytical results for *A_SPP_* were also in good agreement at least in the spectral range 530–700 nm, where this comparison could be made. The SPP wave field could not be separated from the near and radiation field of the nanoantenna in the range 490–530 nm.

The dots in Figure 3 represent the numerically calculated dependence of *A_SPP_* on the radius of the nanoantenna at three different wavelengths and *d* = 10 nm. As evident from the figure, the numerical results agreed in character with the results of the analytic calculations, with a good quantitative agreement observed in the initial portion of the curves, as long as the condition of validity of the dipole approximation *ka* << 1 strictly held [55]. For larger *a*, when the parameter *ka* < 1 (in the case of λ = 550 nm, for example, this corresponded to the range of about 30 to 70 nm in Figure 3), the discrepancy between the analytical and numerical results increased, but remained within 30–40%. For *ka* > 1, the discrepancy rose sharply.

To clarify the reasons for the difference in numerical and analytical results, let us use, instead of the approximate dipole moment of the sphere in a vacuum ($\left|\mu_{a}p_{0}^{sph}\right|$ in Equation (4)), its exact value given by $a_{1}\left(ka\right)/k^{3}$, where $a_{1}\left(ka\right)$ is the “dipole” Mie expansion coefficient. The corresponding analytic dependence calculated at λ = 550 nm is shown as the dashed curve in Figure 3. At *ka* ≲ 1, the discrepancy with the numerical results remains the same and slightly decreases at *ka* > 1. Therefore, the reason for the discrepancy is only to a small extent related to the inaccuracy of calculating the “vacuum” dipole moment of the sphere, and is mainly due to the effect of the substrate. It is also easy to verify that the correction factor *μ_SDE_* that takes into account the “surface dressing effect” in the dipole approximation improved significantly the agreement between the analytical and numerical results. This indicated that the source of the mismatch was the effect of higher order multipole moments, which was not taken into account in the analytical calculations and which became increasingly significant as the radius of the sphere increased in comparison with the width of the gap.

The numerically calculated dependences *A_SPP_(d)* obtained for *a* = 30 nm at three different wavelengths are shown with dots in Figure 4. As seen from the figure for very small gaps d, the numerical results at λ = 550 nm and λ = 600 nm indicated, in contrast to the analytic curves, a very rapid increase in the magnitude of *A_SPP_* (while the gap was smaller than, respectively, ~3 nm and ~1.5 nm). This difference could also be explained by the effect of the images of higher order multipoles arising in the film, which was very “short-range”, and therefore manifested itself only at small (relative to the radius of the sphere) gaps. It should also be taken into account that the excitation efficiency of local plasmon modes of higher orders in the nanoantenna (quadrupole, octopole, etc.), as a rule, dropped sharply with an increase in the wavelength/sphere radius ratio [56]. The influence of the multipoles was, therefore, readily observed for the short-wavelength (λ = 550 nm and λ = 600 nm) dependences and was much less noticeable for the longer-wavelength dependence at λ = 650 nm. The numerical dependences then demonstrated a sharp decrease in the SPP excitation efficiency as the gap increased up to the value *d_SDE_*, which was the same as that for the analytical curves. The numerical results, however, exhibited larger values of *A_SPP_* than the analytical ones in this region, which could also be explained by neglecting higher order multipoles in the analytic calculations. At *d* > *d_SDE_*, as can be seen from the figure, the numerical and analytical dependences were in good agreement.

## 4. Conclusions

Thus, we have shown that the amplitude of the SPP wave excited by a spherical nanoantenna on a plane metal-dielectric interface can be analytically calculated in full 3D geometry with the same method as that used in the waveguide theory for computing guided-mode amplitudes in the presence of current sources. The method yielded a fair agreement with the results of the rigorous numerical simulation when the dipole approximation remained applicable for the nanoantenna. The developed method was shown to provide reasonably accurate results for spherical gold nanoantennas with radii of up to ~70 nm. The presented approach did not require the calculation of Sommerfeld integrals and could be easily extended to the case of an arbitrary current density distribution. We believe that our method can find wide application in modeling diverse phenomena involving SPP excitation.

## Data Availability

Data sharing not applicable.

## Appendix A

The total electric field created by the oscillating point dipole p located at point $r=r_{0}=\left\{x_{0},y_{0},z_{0}\right\}$ in free space can be expressed via the Green’s function $\hat{G_{0}}$ [55] as

(A1) $$E^{d}=\omega^{2}\mu_{0}\hat{G_{0}}\left(r,r_{0}\right)p,$$

where ω is the angular frequency, $\mu_{0}$ is the vacuum permeability, and $\hat{G_{0}}$ is given by the relations

(A2) $$\hat{G_{0}}=\frac{i}{8\pi^{2}}\iint_{-\infty }^{\infty }\hat{M}e^{i\left(k_{x}\left|x-x_{0}\right|+k_{y}\left(y-y_{0}\right)+k_{z}\left(z-z_{0}\right)\right)}dk_{y}dk_{z},$$

(A3) $$\hat{M}=\frac{1}{k_{0}^{2}k_{x1}}\left(\begin{array}{ccc} k^{2}-k_{x1}^{2} & \mp k_{x1}k_{y} & \mp k_{x1}k_{z}\\ \mp k_{x1}k_{y} & k^{2}-k_{y}^{2} & -k_{y}k_{z}\\ \mp k_{x1}k_{z} & -k_{y}k_{z} & k^{2}-k_{z}^{2} \end{array}\right).$$

k=ω/c in the equation above is the vacuum wavenumber, the upper signs correspond to the area with $x>x_{0}$, and the lower signs correspond to the area with $x<x_{0}$.

Below, we focus on the X component of the electric field only. Assuming the Z oriented dipole $p=p_{A}e_{z}=\left\{0,0,p_{A}\right\}$ located at the point $r_{0}=\left\{x_{0},0,0\right\}$, one can express the X component of the electric field in the area with $x<x_{0}$ of free space as

(A4) $$E_{x}^{d}=\omega^{2}\mu_{0}\frac{i}{8\pi^{2}k_{0}^{2}}p_{A}\iint_{-\infty }^{+\infty }k_{z}e^{i\left(-k_{x1}\left(x-x_{0}\right)+k_{y}y+k_{z}z\right)}dk_{y}dk_{z}.$$

Therefore, the X component of the reflected field, which is the only term containing the SPP field can be written as

(A5) $$E_{x}^{d-r}=\omega^{2}\mu_{0}\frac{i}{8\pi^{2}k_{0}^{2}}p_{A}\iint_{-\infty }^{+\infty }k_{z}R\left(k_{y},k_{z}\right)e^{ik_{x1}x_{0}}e^{i\left(k_{x1}x+k_{y}y+k_{z}z\right)}dk_{y}dk_{z},$$

where

(A6) $$R\left(k_{y},k_{z}\right)=\frac{\varepsilon_{1}k_{x2}-\varepsilon_{2}k_{x1}}{\varepsilon_{1}k_{x2}+\varepsilon_{2}k_{x1}}$$

is the Fresnel reflection coefficient at the air/medium interface, index 1 corresponds to the upper half-space, index 2 to the lower half-space, ε is the permittivity of the media, and $k_{\rho }$ is the in-plane wavenumber.

Turning from Cartesian coordinates to cylindrical coordinates and integrating over the polar angle, the X component of the reflected field can be expressed as

(A7) $$E_{x}^{SPP}=-\frac{\omega^{2}\mu_{0}p_{A}}{8\pi k_{0}^{2}}\cos\theta \;p.v.\int_{-\infty }^{+\infty }dk_{r}k_{r}^{2}R\left(k_{r}\right)e^{ik_{x1}\left(x+x_{0}\right)}H_{1}^{\left(1\right)}\left(k_{\rho }r\right),$$

where $r=\sqrt{y^{2}+z^{2}}$, and $H_{1}^{\left(1\right)}$ is the Hankel function of the first kind.

Next, using the Cauchy theorem, one can evaluate the integral:

(A8) $$E_{x}^{SPP}=-\frac{\omega^{2}\mu_{0}p_{A}}{8\pi k_{0}^{2}}\cos\theta \;k_{\mathrm{SPP}}^{2}e^{ik_{x1}\left(x+x_{0}\right)}H_{1}^{\left(1\right)}\left(k_{\mathrm{SPP}}r\right)\times 2\pi i\underset{k_{r}=k_{\mathrm{SPP}}}{\mathrm{Res}}R\left(k_{r}\right),$$

where $k_{\mathrm{SPP}}=k_{0}\sqrt{\varepsilon_{1}\varepsilon_{2}/\left(\varepsilon_{1}+\varepsilon_{2}\right)}$, $k_{x1}$ is evaluated for $k_{r}=k_{\mathrm{SPP}}$, and Res stands for residue.

Finally, after substituting the explicit expression for the residue, one obtains:

(A9) $$E_{x}^{SPP}=-2A_{SPP}\varepsilon_{Me}^{\frac{1}{2}}e^{-\gamma_{D}x}H_{1}^{\left(1\right)}\left(k_{SPP}r\right)\cos\theta$$

with

(A10) $$A_{SPP}=p_{A}k_{SPP}\gamma_{D}^{2}{\left(4\varepsilon_{0}\right)}^{-1}\left(\varepsilon_{Me}^{3/2}/\left(\varepsilon_{Me}^{2}-1\right)\right)\exp(-\gamma_{D}x_{0}).$$

The obtained expression agrees with the results expressed by relation (3).

## Appendix B

Here, our goal is to obtain the efficiency for SPP excitement by considering the Lorentz reciprocity theorem [52] with fields represented by their cylindrical eigenfunction expansions. We consider $\left\{E_{1},H_{1}\right\}$ and $\left\{E_{2},H_{2}\right\}$ to be solutions for the same dielectric-metal structure with the current densities given by $j_{1}=0$ and $j_{2}=-i\omega p\delta \left(r-r_{0}\right)$, where δ(r)=δ(x)δ(y)δ(z) is the Dirac delta function. In such conditions, the Lorentz reciprocity theorem states that

(A11) $$\oint_{\partial V}\left(E_{1}\times H_{2}-E_{2}\times H_{1}\right)dS=i\omega pE_{1}\left(r_{0}\right).$$

for any volume V containing the point $r=r_{0}$.

In the region x>0, SPP fields can be written as [38]

(A12) $$E_{\mathrm{SPP}}=\left(\begin{array}{c} E_{\mathrm{SPP},r}\\ E_{\mathrm{SPP},\theta }\\ E_{\mathrm{SPP},x} \end{array}\right)=\sum_{m}a_{m}\left(\begin{array}{c} \frac{ik_{x1}}{k_{\mathrm{SPP}}}\left(Z_{m+1}\left(k_{\mathrm{SPP}}r\right)-Z_{m-1}\left(k_{\mathrm{SPP}}r\right)\right)\\ \frac{ik_{x1}}{k_{\mathrm{SPP}}}\left(Z_{m+1}\left(k_{\mathrm{SPP}}r\right)+Z_{m-1}\left(k_{\mathrm{SPP}}r\right)\right)\\ 2Z_{m}\left(k_{\mathrm{SPP}}r\right) \end{array}\right)e^{ik_{x1}x+im\theta },$$

(A13) $$H_{\mathrm{SPP}}=\underset{m}{\sum }a_{m}\frac{\omega \varepsilon_{0}\varepsilon_{1}}{k_{\mathrm{SPP}}}\left(\begin{array}{c} Z_{m+1}\left(k_{\mathrm{SPP}}r\right)+Z_{m-1}\left(k_{\mathrm{SPP}}r\right)\\ -\left(Z_{m+1}\left(k_{\mathrm{SPP}}r\right)-Z_{m-1}\left(k_{\mathrm{SPP}}r\right)\right)\\ 0 \end{array}\right)e^{ik_{x1}x+im\theta },$$

where $Z_{m}$ could be any cylindrical function of order m (in particular, in the Hankel function of the first kind $Z_{m}=H_{m}^{\left(1\right)}$ corresponds to the outgoing SPP, and in the Hankel function of the second kind $Z_{m}=H_{m}^{\left(2\right)}$ corresponds to the incoming SPP), $k_{\mathrm{SPP}}=k_{0}\sqrt{\varepsilon_{1}\varepsilon_{2}/\left(\varepsilon_{1}+\varepsilon_{2}\right)}$, $k_{x1}=i\sqrt{k_{\mathrm{SPP}}^{2}-\varepsilon_{1}k_{0}^{2}}=i\gamma_{D}$, $\varepsilon_{1}=1$, and $k_{0}=\omega /c$. Therefore, the relations for the electric and magnetic fields can be sought in the form

(A14) $$E_{1}=\underset{m}{\sum }A_{m}\left(\begin{array}{c} \frac{ik_{x1}}{k_{\mathrm{SPP}}}{J_{m}}^{\prime }\left(k_{\mathrm{SPP}}r\right)\\ \frac{mk_{x1}}{k_{\mathrm{SPP}}^{2}r}J_{m}\left(k_{\mathrm{SPP}}r\right)\\ J_{m}\left(k_{\mathrm{SPP}}r\right) \end{array}\right)e^{i\left(k_{x1}x-m\theta \right)}=\underset{m}{\sum }A_{m}E_{m}^{-},\;$$

(A15) $$H_{1}=\underset{m}{\sum }A_{m}\frac{\omega \varepsilon_{0}\varepsilon_{1}}{k_{\mathrm{SPP}}}\left(\begin{array}{c} -\frac{m}{k_{\mathrm{SPP}}r}J_{m}\left(k_{\mathrm{SPP}}r\right)\\ i{J_{m}}^{\prime }\left(k_{\mathrm{SPP}}r\right)\\ 0 \end{array}\right)e^{i\left(k_{x1}x-m\theta \right)}=\underset{m}{\sum }A_{m}H_{m}^{-}$$

(A16) $$E_{2}=\tilde{E_{2}}+\underset{m}{\sum }B_{m}\left(\begin{array}{c} \frac{ik_{x1}}{k_{\mathrm{SPP}}}{H_{m}^{\left(1\right)}}^{\prime }\left(k_{\mathrm{SPP}}r\right)\\ -\frac{mk_{x1}}{k_{\mathrm{SPP}}^{2}r}H_{m}^{\left(1\right)}\left(k_{\mathrm{SPP}}r\right)\\ H_{m}^{\left(1\right)}\left(k_{\mathrm{SPP}}r\right) \end{array}\right)e^{i\left(k_{x1}x+m\theta \right)}\;=\tilde{E_{2}}+\underset{m}{\sum }B_{m}E_{m}^{+}$$

(A17) $$H_{2}=\tilde{H_{2}}+\underset{m}{\sum }B_{m}\frac{\omega \varepsilon_{0}\varepsilon_{1}}{k_{\mathrm{SPP}}}\left(\begin{array}{c} \frac{m}{k_{\mathrm{SPP}}r}H_{m}^{\left(1\right)}\left(k_{\mathrm{SPP}}r\right)\\ i{H_{m}^{\left(1\right)}}^{\prime }\left(k_{\mathrm{SPP}}r\right)\\ 0 \end{array}\right)e^{i\left(k_{x1}x+m\theta \right)}\;=\tilde{H_{2}}+\underset{m}{\sum }B_{m}H_{m}^{+},$$

where $J_{m}\left(x\right)$ stands for the Bessel function of order m and the tilde terms do not contain outcoming surface waves.

Expressions for the region x<0 can be obtained from (A14)–(A17) by replacement of the permittivity of the dielectric media $\varepsilon_{1}$ by the permittivity of the metal substrate $\varepsilon_{2}=\varepsilon_{Me}$, $k_{x1}$ by $k_{x2}=-i\sqrt{k_{\mathrm{SPP}}^{2}-\varepsilon_{2}k^{2}}=-i\gamma_{Me}$, and multiplication of $E_{m}^{\pm }$ and $H_{m}^{\pm }$ by $\frac{k_{x1}}{k_{x2}}$ in order to satisfy the interface conditions.

Considering the integration volume V as an infinitely long cylinder oriented along the X axis, one can rewrite (A11) as

(A18) $$\int_{0}^{2\pi }rd\theta \;\int_{-\infty }^{+\infty }dx\left(E_{1}\times H_{2}-E_{2}\times H_{1}\right)e_{r}=i\omega pE_{1}\left(r_{0}\right).\;$$

Due to the orthogonality of the different modes, the unknown coefficient $B_{m}$ can be calculated as

(A19) $$B_{m}=\frac{i\omega pE_{m}^{-}\left(r_{0}\right)}{N_{m}},\;$$

where $N_{m}$ is given by

(A20) $$N_{m}=\int_{0}^{2\pi }r\;d\theta \int_{-\infty }^{+\infty }dx\left(E_{m}^{-}\times H_{m}^{+}-E_{m}^{+}\times H_{m}^{-}\right)e_{r}\;=\frac{2k_{x1}}{\omega^{2}\mu_{0}k_{\mathrm{SPP}}^{2}}\frac{\left(\varepsilon_{2}^{2}-\varepsilon_{1}^{2}\right)\left(\varepsilon_{1}+\varepsilon_{2}\right)}{\varepsilon_{1}^{2}\varepsilon_{2}^{2}}.\;$$

For the point dipole $\left|p\right|=p_{A}$ located at $r_{0}=\left(0,0,x_{0}\right)$ which is parallel to the interface, there are only two azimuthal numbers m supporting the nonzero product:

(A21) $$pE_{\pm 1}^{-}=\pm \frac{ik_{x1}}{2k_{\mathrm{SPP}}}p_{A}e^{ik_{x1}x_{0}\mp i\theta }.\;$$

Consequently, the electric field of the outgoing SPP in the upper half-plane can be expressed as

(A22) $$E_{x}^{SPP}=-2A_{SPP}\varepsilon_{Me}^{\frac{1}{2}}e^{-\gamma_{D}x}H_{1}^{\left(1\right)}\left(k_{SPP}r\right)\cos\theta$$

with

(A23) $$A_{SPP}=p_{A}k_{SPP}\gamma_{D}^{2}{\left(4\varepsilon_{0}\right)}^{-1}\left(\varepsilon_{Me}^{3/2}/\left(\varepsilon_{Me}^{2}-1\right)\right)\exp(-\gamma_{D}x_{0}).$$

The obtained expression agrees with the results expressed by relations (3) and (A10).
